# Sub-trabecular strain evolution in human trabecular bone

**DOI:** 10.1038/s41598-020-69850-x

**Published:** 2020-08-14

**Authors:** Mikael J. Turunen, Sophie Le Cann, Erika Tudisco, Goran Lovric, Alessandra Patera, Stephen A. Hall, Hanna Isaksson

**Affiliations:** 1 Department of Applied Physics, University of Eastern Finland, Box 1627, 70211 Kuopio, Finland; 2 Department of Biomedical Engineering, Lund University, Lund, Sweden; 3 Division of Geotechnical Engineering, Lund University, Lund, Sweden; 4 Swiss Light Source, Paul Scherrer Institute, Villigen, Switzerland; 5 Centre D’Imagerie BioMédicale, École Polytechnique Fédérale de Lausanne, Lausanne, Switzerland; 6 Division of Solid Mechanics, Lund University, Lund, Sweden; 7 Lund Institute of advanced Neutron and X-ray Science (LINXS), Lund, Sweden; 8 Department of Orthopaedics, Clinical Sciences, Lund University, Lund, Sweden

**Keywords:** Bone quality and biomechanics, Bone, Biomedical engineering, Imaging techniques

## Abstract

To comprehend the most detrimental characteristics behind bone fractures, it is key to understand the material and tissue level strain limits and their relation to failure sites. The aim of this study was to investigate the three-dimensional strain distribution and its evolution during loading at the sub-trabecular level in trabecular bone tissue. Human cadaver trabecular bone samples were compressed in situ until failure, while imaging with high-resolution synchrotron radiation X-ray tomography. Digital volume correlation was used to determine the strains inside the trabeculae. Regions without emerging damage were compared to those about to crack. Local strains in close vicinity of developing cracks were higher than previously reported for a whole trabecular structure and similar to those reported for single isolated trabeculae. Early literature on bone fracture strain thresholds at the tissue level seem to underestimate the maximum strain magnitudes in trabecular bone. Furthermore, we found lower strain levels and a reduced ability to capture detailed crack-paths with increased image voxel size. This highlights the dependence between the observed strain levels and the voxel size and that high-resolution is needed to investigate behavior of individual trabeculae. Furthermore, low trabecular thickness appears to be one predictor of developing cracks. In summary, this study investigated the local strains in whole trabecular structure at sub-trabecular resolution in human bone and confirmed the high strain magnitudes reported for single trabeculae under loading and, importantly extends its translation to the whole trabecular structure.

## Introduction

Bone can withstand particularly high loads. For example, at the organ level the human proximal femur can endure up to 10 times the bodyweight^1–4^. Bone strength is provided both by the material and its heterogeneous hierarchical structure in which each length scale has its important role^5^. At the tissue level, the forces are distributed and transferred through the bone based on the relations between the spongy trabecular network and the dense cortical bone. Trabecular bone is metabolically highly active and undergoes remodeling throughout life^6–8^. During aging and when affected by metabolic bone diseases such as osteoporosis, the metabolic activity changes as bone turnover is increased^5, 6, 9^. These alterations result in net-loss of bone tissue, with decreased structural connectivity and apparent density, which subsequently leads to a weaker structure. To understand the most detrimental characteristics behind bone fractures, it is important to investigate the fracture initiation and find the relationships between failure sites and measurable bone parameters. Many studies utilize computational finite element analysis to investigate the local bone strains during loading^10–12^. However, such studies are restricted because of limited experimental data available for validation. The lack of direct experimental measurements of the evolution of strains in bone during loading until failure motivate the need for high-resolution image-based methods and experiments.

Loads and resulting strains in bone are transferred through the trabecular tissue, which has been optimized to the local mechanical loading demands through remodeling^13^. Locally, the primary orientation of the trabeculae is aligned with the global main force direction^7^, while secondary oriented trabeculae complete the optimized network of the tissue^14^. The forces deform the trabecular structure in such a way that individual trabeculae endure strains that can be compressive, tensile, or in shear, depending on the direction of the load and the trabecular network. At the organ scale, bone mineral density (BMD) is an important contributor to overall strength and fracture toughness, while the contribution of the local micro-scale tissue mineral density (TMD) and structural variations on crack resistance remains unknown. Hypothetically, there are two factors that can directly affect the location of the failure at the trabecular level: (1) the trabecular microstructure and organization, *i.e.* the amount, connectivity, and orientation of the trabecular bone network, and (2) the local material properties, e.g. TMD. Earlier studies have investigated their effects on the resulting strains in trabecular bone by considering the structure of the trabecular network^10–12, 15^ or on isolated single trabeculae^16–21^, respectively.

In general, earlier studies considering the structure of the trabecular bone network suggest that compressive loading causes global yielding at ~ 1 to 4% local tissue strain^10–12, 15^. These findings are largely based on finite element computational models^10–12^, but also plane 2D digital images^15^. The underlying isotropic image voxel size used for such models ranged between 20 and 63 µm. Nagaraja et al. reported between 1 and 2.4% local von Mises strains in trabeculae at 1.5% global strain (corresponding approximately to the yield point) in bovine trabecular bone under uniaxial compression^10^. Morgan et al. reported maximum principal strains of 1–4% in damage regions when using a low (maximum of 0.45%, not reported as yield point) apparent strain multi-cycle loading protocol on trabecular bone from human vertebrae^11^. Bayraktar et al. measured a 0.6–1.0% tissue yield strain at 0.8% global compressive yield strain in human cadaver femoral neck trabecular bone^12^. Odgaard et al. on the other hand concluded that local ultimate strains at a failing part of human proximal tibiae trabecular bone were 3.7% with a respective global strain of 2.7%^15^. Although all the studies cited here report slightly different types of strains to describe yielding of trabecular bone (von Mises, maximum principal, tissue yield, ultimate strains) measured with different setups, the levels are reasonably consistent.

When single trabeculae are subjected to loading, the mechanical properties are mostly affected by the material level properties of bone^16–21^. Surface strain mapping of single trabecula from bovine bone has shown that the strains at initiation of micro-damage and the ultimate strain just before fracture were 1.6% and 12.0%, respectively^16^. Hernandez et al. observed that the ductility of individual trabeculae can vary vastly, based on reported ultimate strains ranging from 1.8 to 20.2% of individual trabeculae tested under tension^20^. Carreta et al. have extensively shown that molecular composition is an important factor for the mechanical properties of trabecular bone, all having high within-subject variability^17–19^. In bovine trabecular bone, they reported ultimate strains in tension ranging between 4.5% and 5.1% and ultimate strains under bending of 16.7–18.4%^18^. In individual human trabeculae, the ultimate strains in tension ranged between 2.4% and 5.1% and in bending from 6.0 to 8.2%^19^. Compared to studies of the whole trabecular structure, strains in these single trabeculae studies are substantially greater. The studies on single isolated trabeculae significantly advanced the understanding of bone damage mechanisms. It has to be noted, that the strains measured in the single trabeculae represent the strain level that trabecular tissue material can withstand on its own before failing. In contrast, in the studies considering the structure of the trabecular bone network^10–12^, except for the study by Odgaard et al.^15^, the yielding strains were measured at a noteworthy point in the samples stress–strain curve and these are thus not fully comparable. It is not granted that the findings from single trabeculae are generalizable to the trabecular network structure level, which experiences large-deformation, bending, and buckling under loading. In this context, novel computational models have been developed to investigate this interconnection^22, 23^, but they lack experimental verification at the correct scale.

Image correlation methods have been used increasingly to analyze tissue strains during in situ loading. These methods can be divided into surface^2, 24^ and volumetric image correlations^25, 26^. The surface scenario is usually referred to as digital image correlation (DIC), whereas the volumetric scenario, an extension of DIC, is generally referred to as digital volume correlation (DVC). DVC is currently the only experimental measurement technique to calculate the 3D distributions of strain magnitudes within a biological structure, which makes it suitable to validate fracture criteria in computational models.

Conducting in situ loading experiments together with tomography enables acquisition of sequences of 3D images of a sample during loading. DVC can then be applied to 3D sub-regions of two image volumes from different stages of deformation, and internal displacements can be calculated by tracking the structures from one load step to another. DVC was first used to study overall internal strain distribution within a whole trabecular bone plug^26^ and most existing work has studied the displacement and strain distributions and magnitudes at the macro or trabecular levels^26–30^. In these studies, the isotropic image voxel size varied between 12 and 39 µm, resulting in DVC sub-volume size of 252–2,200 µm. Since trabecular thickness is on average around 200 µm, such sub-volume sizes are not sufficient to investigate strains within trabeculae. Since the strains in DIC and DVC are calculated from the gradient of the displacement in a displacement field with a given spatial resolution, there is generally a dependence between strain magnitude and image voxel size^31^.

Moreover, extensive work has been done on bone micro-tomograms to investigate the precision and accuracy, local displacement and strain uncertainties, as well as the effect of the volumetric resolution of the DVC procedure^25, 32–36^. Most previous studies have used images acquired with laboratory µCT, which results in high uncertainties related to longer imaging times. From this perspective, it is highly beneficial to use synchrotron radiation micro-computed tomography (SR-µCT)^32, 37^, an imaging technique which has become increasingly popular in bone research^38–40^. In particular, the possible fast imaging times and high resolution (< 0.1 µm voxel size) have led musculoskeletal tissue scientists to synchrotron facilities^37^.

The aims of this study were to investigate the distribution and magnitudes of internal strains at sub-trabecular resolution in human trabecular bone and their evolution during loading. Moreover, the study aims to investigate the relationship between material and tissue level biomechanical properties and to bridge the discrepancies in strain levels when investigated in single trabeculae or in whole trabecular structure. A subsequent aim was to investigate the influence of image voxel size on the strain magnitudes. Finally, this study describes the links between local crack sites, tissue mineral density, trabecular microstructure, and local tissue strains with sub-trabecular resolution.

## Materials and methods

### Sample preparation

**Figure 1:**
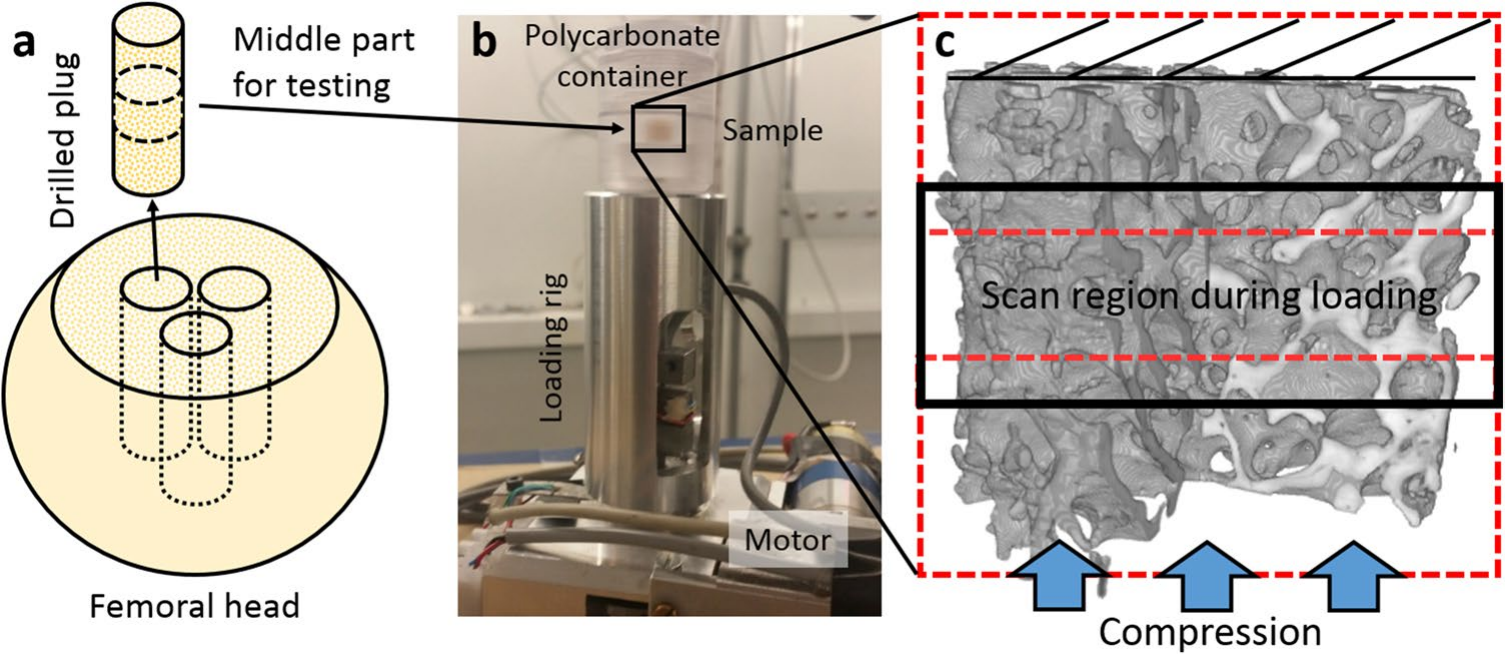
Sample loading and SR-µCT imaging. (**a**) Trabecular bone plugs were collected from human femoral heads using a trephine drill and the middle part of the plugs were sawed off for testing. (**b**) Samples were compressed using a custom-made loading rig while imaging. (**c**) The X-ray scan FOV was smaller in height than the sample, thus only the middle part (black box) was imaged during loading. Both ends with overlapping regions were acquired before and after loading to image the entire plug (red dotted boxes).

Femora were harvested from human cadavers (N = 13, 12 male and 1 female with age span of 21–82 years) with no known metabolic or infectious bone diseases. Permission was obtained from the legally authorized representative (National Authority for Medicolegal Affairs, TEO, 5,783/04/044/07). The samples were collected in Finland. According to Finnish legislation, for scientific studies using cadaver human samples ethical permission is granted by the ethical committee, National Authority for Medicolegal Affairs, and informed consent from next of kin is not required. The femoral heads were sawn off perpendicular to the femoral necks. Cylindrical trabecular bone plugs (height ~ 26 mm, diameter ~ 7.1 mm, n = 1 per femoral head used in this study) were drilled perpendicular to the cut surface, towards the femoral head, using a trephine drill under constant phosphate buffered saline (PBS) irrigation (Fig. 1a). The proximal ends of the cylindrical plugs were sawed perpendicular to the length of the cylinder with a low speed saw (Buehler Isomet, Buehler, IL) resulting in a ~ 6.3 mm long plug (Fig. 1a). The bone plugs were extracted from the same anatomical position in each femoral head to minimize the effects of local variations. After extraction, the plugs were immersed in PBS and frozen in − 20 °C.

### Experimental setup

The experiments were carried out at the X02DA TOMCAT beamline (Swiss Light Source, Paul Scherrer Institut, Switzerland), where the X-rays are produced by a 2.9 T superbending magnet on a 2.4 GeV storage ring (operated in top-up node with ring current up to 400 mA). After thawing, the trabecular bone plugs were placed inside the polycarbonate container of a custom-made loading rig (Fig. 1b) and imaged under zero-load in three overlapping regions (Fig. 1c). The field of view (FOV) height was ~ 2.7 mm, covering only part of the sample, and ~ 7.1 mm in width, including the whole sample width. Due to the height of the FOV, only the middle part of the sample was imaged during loading (Fig. 1c). The samples were imaged using a monochromatic X-ray beam with an energy of 30 keV. A high-speed CMOS (Complementary Metal Oxide Semiconductor) detector (pco.Dimax) coupled to visible-light optics with a 100 µm thick scintillator (LuAg:Ce) was used. A full tomographic dataset consisting of 1801 projections (over 180 degrees) and dark and flat fields was acquired in 45 s. The voxel size was 3.6 × 3.6 × 3.6 µm^3^ with continuously changeable magnifying optics^41^, which was sufficient to visualize sub-trabecular structures. Immediately after the unloaded scans, the samples were compressed in displacement rate controlled steps and the loading was stopped to scan the sample in steady-state conditions, minimizing movement artifacts during image acquisition. The loading was resumed immediately after each scan and this procedure was repeated until fracture (when the load–displacement curves showed clear post-yield behavior). After failure, the whole plug was scanned again in three overlapping regions. The number of load steps (and thus tomography scans) per sample varied between 4 and 9 depending on when failure was reached. The sample was in a closed loading container for the whole measurement protocol, which lasted a maximum of 25 min (depending on the number of scans) after removing the sample from the PBS immersion. No significant dehydration was observed. Additionally, a set of hydroxyapatite phantoms (250, 750, and 1,250 mg/cc) were imaged to calibrate the image grayscale values to volumetric tissue mineral density (TMD) values.

Utilization of SR-µCT exposes the samples to a large radiation dose, which can be detrimental to the sample constituents, especially the collagen, and may affect the mechanical properties of the samples^42–44^. Therefore, a simplified dose model for the given acquisition settings was adopted to estimate the total absorbed dose for each tomographic scan^45^. Namely, the samples were modeled as binary structures consisting of adipose tissue (density of 0.9) and bone (density of 1.32) with a mixture of fractions of 0.38–0.51 (fat) versus 0.49–0.62 (bone) depending on the bone volume fraction (BV/TV) of the sample. The mass attenuation coefficients were adapted from the NIST database and the X-ray flux was measured independently with passivated, implanted planar silicon (PIPS) diodes^41^. This resulted in an estimated total radiation dose (per sample) between 9 and 20 kGy, mainly depending on the number of scans per sample. Moreover, each sample was subjected to additional radiation dose during positioning/alignment, approximated to be < 2.5 min per sample, thus adding a maximum of ~ 8 kGy to the estimated doses.

Tomographic image reconstructions were performed using the gridrec algorithm^46^ with fixed parameters for all data sets to enable quantitative comparison of the densities. Prior to reconstruction, the projections were corrected and normalized with the flat-field and dark-field images^46^.

### Microstructure and tissue mineral density analysis

**Figure 2:**
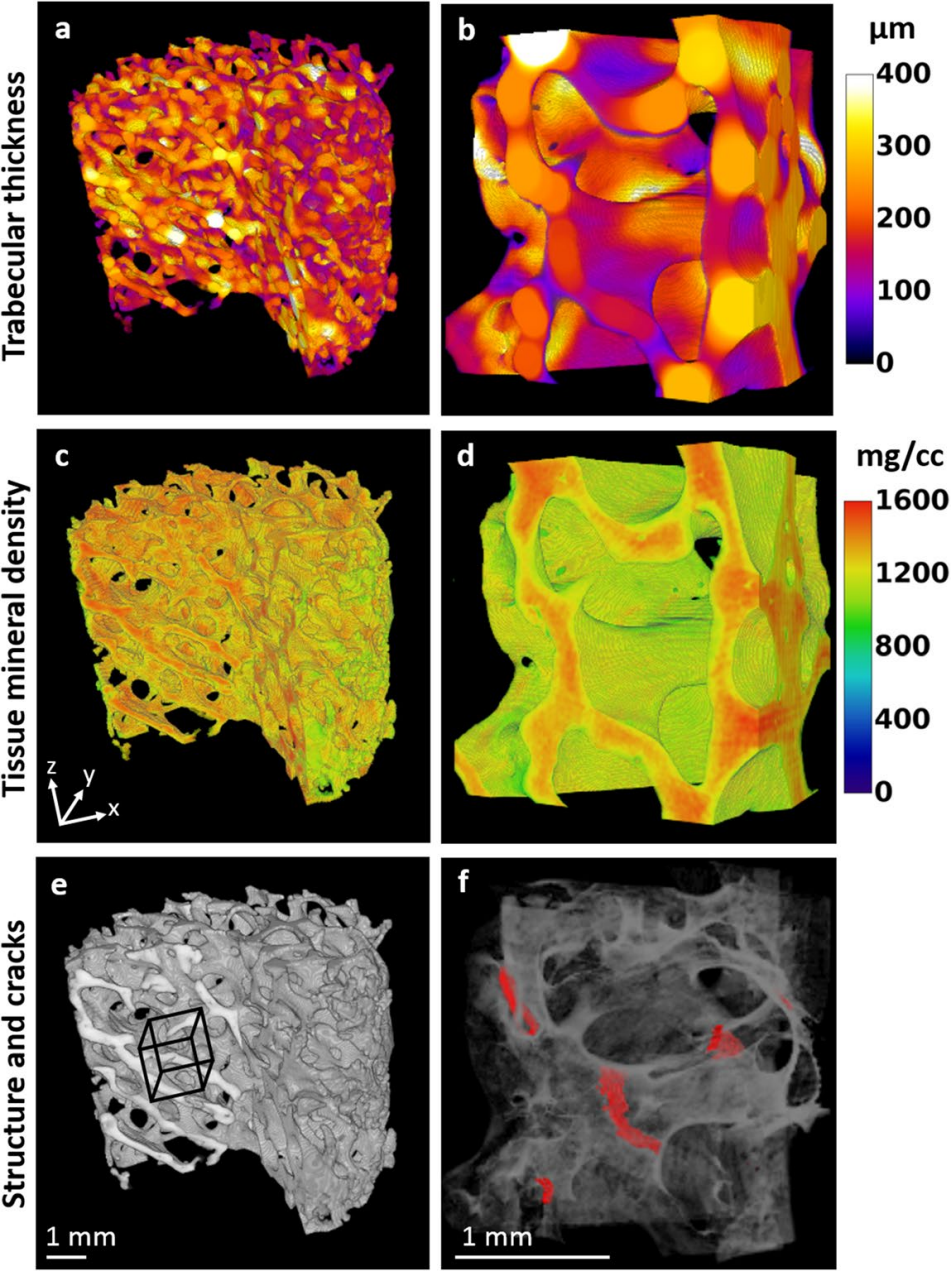
Role of trabecular thickness and tissue mineral density to the occurrence of cracks. 3D maps (sample 8) to visualize variation within the structure of (**a**, **b**) trabecular thickness, (**c**, **d**) mineral density, and (**e**, **f**) grayscale trabecular structure, with cracks highlighted in red. The right column represents the sub-region used in the DVC analysis, of dimensions 2.17 × 2.17 × 1.45 mm^3^ (x, y, z). The location and size of the sub-region is shown in (**e**). The trabecular thickness maps (**a**, **b**) were created with BoneJ^47^ (ImageJ 1.52p, https://imagej.net/) and the mineral density maps (**c**, **d**) with custom made MatLab scripts (R2017b, The MathWorks Inc., MA) (see ‘Microstructure and tissue mineral density analysis’). The crack regions (**f**) were identified and segmented using custom made MatLab scripts (see “Crack identification and segmentation”). All subfigures were plotted using ImageJ 3D Viewer (https://imagej.net/3D_Viewer).

The initial microstructure and tissue mineral density of the samples were determined from the images of the entire sample acquired before loading, obtained by stacking the three overlapping images. The microstructure was analyzed using BoneJ^47^ (ImageJ 1.52p). A grayscale threshold value with range 115–255 (8-bit image) was assigned to mineralized bone tissue, corresponding to 833–1746 mg/cc mineral density. For each sample, the trabecular BV/TV, trabecular thickness (Tb.Th.), trabecular number (Tb.N.), and degree of anisotropy (DA) were determined according to standard nomenclature^48, 49^. Moreover, trabecular thickness maps were created using BoneJ (Fig. 2a, b). TMD maps were generated (custom-made MatLab script) based on the calibration curve obtained from the phantoms, where each voxel represents the local density value (Fig. 2c, d).

### Compressive loading and analysis

**Figure 3:**
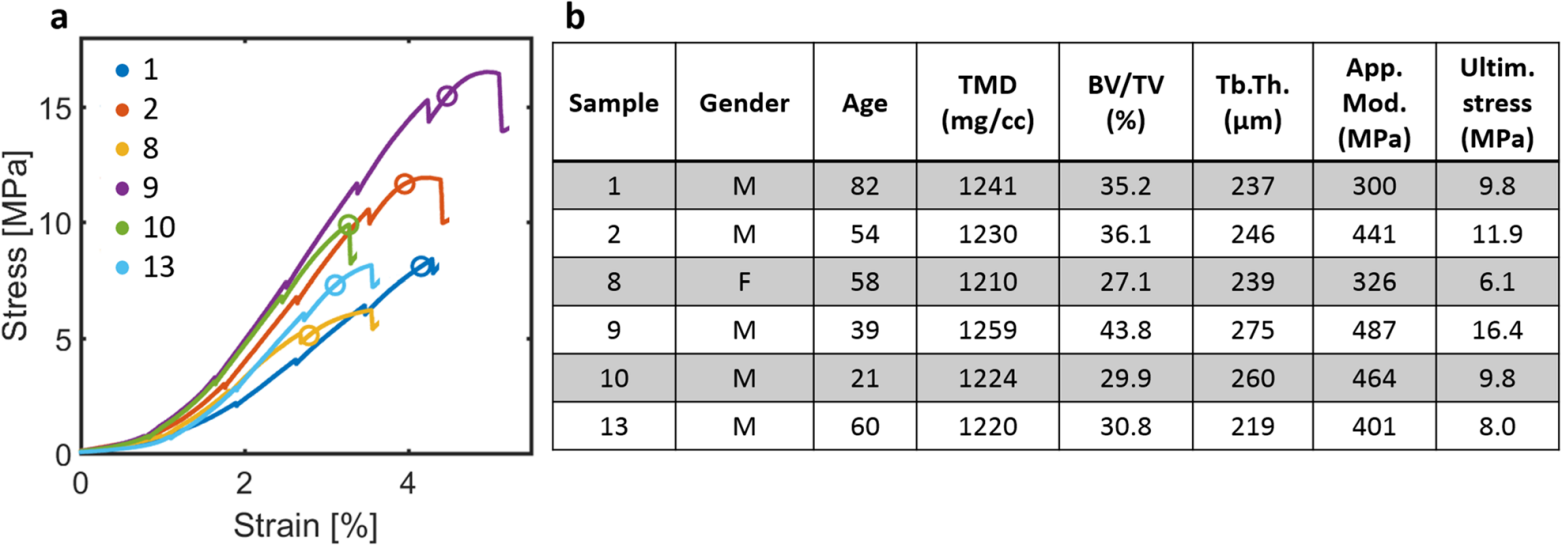
Loading curves and characteristics of samples used for DVC analysis. (**a**) Stress–strain compressive loading curves until ultimate stress for each sample. The drops in the curves are due to the relaxation of the sample while stopping the loading during imaging. Open circles indicate the yield points. (**b**) Samples’ characteristics such as gender, age, sample tissue mineral density (TMD), bone volume fraction (BV/TV), trabecular thickness (Tb.Th.), apparent modulus (App. Mod.), and ultimate stress (Ultim. stress).

Using a custom-made loading rig, load–displacement data were recorded throughout the experiment for each sample. From the load–displacement data, stress–strain curves were calculated for the whole sample based on the dimensions of the samples (Fig. 3a). The displacement values were corrected to account for the compliance of the loading system, obtaining the total displacement using the scans before and after loading. Each loading step was ~ 0.85% with ~ 0.85%/min loading rate after accounting for the system compliance. Bulk mechanical parameters were determined from the loading curves, such as apparent modulus, ultimate stress, and toughness. The apparent moduli were determined from the linear region of the stress–strain curves (excluding the pauses in loading) and toughness was calculated as the area under the stress–strain curve until the point of ultimate stress. Additionally, using the 0.2% strain offset method, yield stress was determined. Due to minor imperfections in contact between the sample and the loading piston during the pre-load (30 N), i.e. a possible small tilt and surface roughness of the boundaries of the bone sample plugs, a toe-region was evident during early loading of the samples (Fig. 3a). Instead of choosing a higher pre-load, the ultimate strain (%) and yield strain (%, the strain corresponding to the yield stress) were determined as *delta strain* excluding the toe-region of the loading curve by extrapolating the linear part of the curve to zero-stress and by subtracting this value from the raw values. Data analysis was performed using custom MatLab scripts (R2017b, The MathWorks Inc., MA). From all the samples, a subset of six samples that differed in gender, age, trabecular bone microstructure, and mechanical parameters, were chosen for detailed DVC analysis (Fig. 3b).

### Digital volume correlation analysis

Image stacks between consecutive loading steps were pre-processed using custom-made MatLab scripts before DVC analysis. This involved aligning the images for rigid deformation, filtering using a 3D median filter of ± 3 voxels to reduce noise and masking of voids/background, which were not included in the correlation analysis (Supplementary Fig. 1). Due to the synchrotron µCT measurement setup, e.g. fast scanning time, the acquired images at different phases of loading contained variable noise. The selected 3D median filter was adopted to remove noise while keeping the internal density variation within the tissue.

A region of interest (601 × 601 × 401 voxels, i.e. 2.17 × 2.17 × 1.45 mm^3^) where cracked trabeculae were observed by visual inspection (Fig. 2e, f) was isolated in all loaded scans for each sample and analyzed using a local DVC approach with the Python-based TomoWarp2 code. A detailed description of the software can be found in Tudisco et al.^50^. Briefly, the displacement fields were obtained by tracking the translation of small sub-volumes (i.e. correlation windows, CW) between two subsequent loading steps. Sub-pixel refinement was first performed by finding the maximum of a tri-quadratic function interpolating the correlation coefficient at the 27 points surrounding the best integer-displacement. For nodes that could not be resolved with this method (i.e. where no maximum of the interpolating function was found within the local neighborhood), the sub-pixel displacements were instead determined by a numerical procedure. This procedure involved resampling (using tricubic interpolation) of the sub-volume of the deformed image around the node for sub-pixel displacements within a gradient-based optimization scheme to converge on a match with the respective reference sub-volume. The optimal correlation window size was found from testing, and was ± 10 voxels and with a node spacing (NS) of 10 voxels, it yielded a 50% overlap. Consequently, displacement maps were derived over a regular grid with a NS corresponding to 10 voxels in the original tomography images. These fields were filtered by removing outliers (where a point was considered to be an outlier when its values diverged more than double from the median value of the surrounding points) and a 3D median filter (R = 1). The Green strain tensor field was then calculated from the filtered displacement fields using linear shape functions on 8-node isoparametric hexahedron finite elements whose nodes correspond to the grid points in the displacement field. The volumetric strains were calculated from det(F)-I, where F is the deformation gradient. Depending on the local tissue volume change, the volumetric strain values were either positive (tensile strain, i.e. volumetric expansion, in the used coordinate system) or negative (compressive strain, i.e. volumetric reduction).

DVC analysis was also performed on downscaled SR-µCT scans to evaluate the effect of image voxel size on strain magnitudes. The filtered scans were downscaled by a factor of 4 and 8 resulting in an isotropic voxel size of 14.44 µm and 28.88 µm, respectively. For the DVC analysis, a CW of ± 7 voxels and NS of 7 voxels were chosen for images downscaled by 4, and CW ± 5 voxels, and NS = 5 voxels were used for images downscaled by 8. Otherwise, the DVC protocol was identical for high-resolution and downscaled data.

Accuracy and precision of the calculated strains (Table 1) were evaluated for each sample from the non-loaded high-resolution and downscaled image stacks that were virtually shifted 2 voxels in all three orthogonal directions. Also, accuracy and precision were evaluated from overlapping regions of the stacked (repeated) scans. The same DVC analysis parameters and protocol as applied to the loaded images were used. Accuracy was defined as the mean and precision as the standard deviation (SD) of the average of the absolute values of the six components of strain in each node^34^, only voxels with a correlation coefficient greater than 0.95 were included.

**Table 1:** Accuracies and precisions for DVC analysis on high-resolution (HR) and downscaled (DS4 and DS8) data analyzed from the virtually shifted and repeated scans. Mean ± SD are shown.

	Virtually shifted		Repeated scans	
	Accuracy	Precision	Accuracy	Precision
HR	597 ± 20 µε (~ 0.06%)	290 ± 15 µε (~ 0.03%)	1638 ± 138 µε (~ 0.16%)	934 ± 97 µε (~ 0.09%)
DS4	1,148 ± 47 µε (~ 0.11%)	478 ± 21 µε (~ 0.05%)	5,294 ± 1,473 µε (~ 0.53%)	4,973 ± 2050 µε (~ 0.50%)
DS8	1706 ± 102 µε (~ 0.17%)	827 ± 81 µε (~ 0.08%)	3,135 ± 675 µε (~ 0.31%)	2068 ± 1,023 µε (~ 0.21%)

### Crack identification and segmentation

The crack regions in the DVC sub-regions were determined for each sample from the final scan (after global yield) of each sample (Fig. 2f) using two consecutive steps: (1) a custom-made automated crack identification and segmentation algorithm followed by (2) a manual inspection (both in MatLab). A crack was defined as a visible discontinuity in the structure in the last scan, which was not present in earlier scans. The final segmentation was based solely on visual inspection. Although the automated crack identification and segmentation algorithm was used only as a tool to aid the final visual segmentation, it is introduced here briefly.

First, an automated segmentation based on thresholding and binary operations was realized using custom-made MatLab scripts. Using a grayscale threshold to mask the voids, *i.e.* to keep the bone tissue (pores and cracks included), the images were binarized (BS1). The edges were detected (MatLab inbuilt function edge3) and dilated (imdilate) with a radius of 12 voxels (BS1e). Next, a second binarized stack (BS2) was created using a higher threshold to mask the regions that were not bone tissue, *i.e.* removing the cracks and the pores within the trabeculae. To visualize only the cracks (BSc), the stacks were subtracted as BSc = BS1–BS2–BS1e, and any voxels with a negative value (because of the subtraction) were set to zero. This automated part of the procedure provided suggestions of cracks (value of 1 in binary image) that sped up the segmentation process.

As the automated procedure also captured canals within the trabeculae and did not reveal cracks that had low contrast compared to the surrounding bone or cracks that were too small, the automated segmentation was corrected manually. The manual correction included removal of falsely detected cracks (e.g. canals) and addition of undetected cracks by comparing visually the corrected image stack with cracks with an image stack at an earlier time-point to ensure that the cracks were not present before. Finally, the corrected BSc was overlaid on the original stack to mark the crack regions (Fig. 2f).

### DVC strain analysis

To investigate the influence of local volumetric strains, trabecular thickness, and TMD on the occurrence of cracks, the accumulation of strains during loading was investigated in (1) *crack regions* (identified from the post-yield images as described in “Crack identification and segmentation”), and (2) *non-crack regions*, i.e. the remainder of the tissue that was not classified as the crack region (based on crack segmented stacks in “Crack identification and segmentation”). The crack regions included the segmented cracks plus 5 voxels, i.e. ~ 18 µm, in all directions from the crack. This region was determined using 3D-dilation of the binary images of the crack regions.

**Figure 4:**
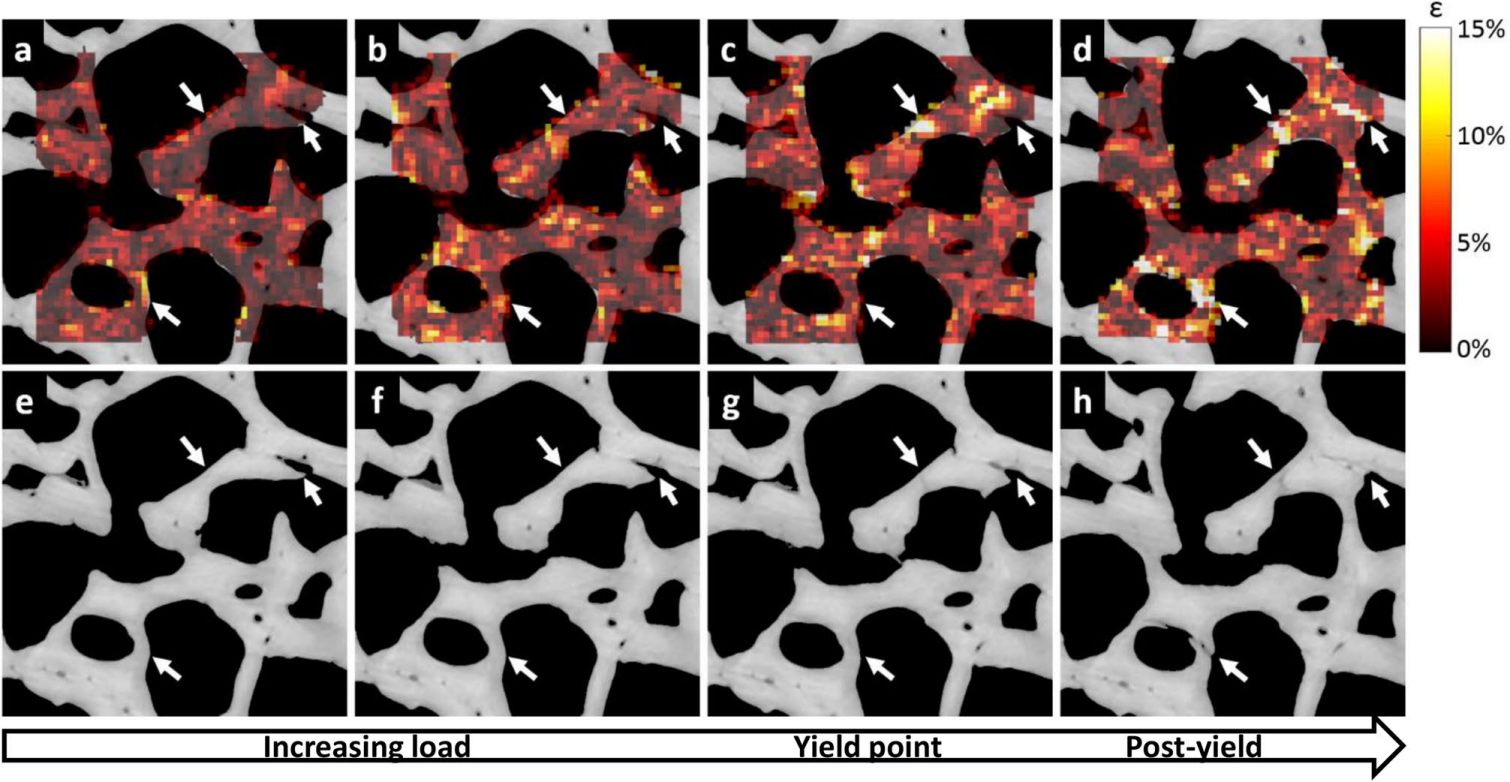
Evolution of strain distribution within trabecular bone during compression. Local absolute cumulative volumetric strain distributions superimposed on each respective SR-µCT section of sample 1 at (**a**) 3rd loading step, (**b**) 4th, (**c**) 5th, and (**d**) 6th (used for crack segmentation). (**e**–**h**) Respective SR-µCT sections without overlaying of the strains. The white arrows indicate locations of occurring cracks. Note that some individual points in the bone exceed the average strains without always resulting in a (visible) crack. The global yield point for this sample is around step (**c**) (or (**g**)). The direction of loading is perpendicular to the figure plane.

The DVC strain analysis was performed only on images from load-steps before reaching global yield, thus when no cracks were visually observed. Only strains from nodes with a correlation coefficient greater than 0.95 were included in the analysis. The DVC strain maps were linearly interpolated to fit the voxel size of the TMD and trabecular thickness maps to enable comparison of the same regions. Cumulative volumetric strains were calculated for each voxel by summing strains from every loading step voxel-by-voxel (Fig. 4). Each voxel at each step had a negative (compressive strain) or positive (tensile strain) volumetric strain value (Supplementary Fig. 2). Strain values after global yield or in regions where a crack was visible were not included in the analysis. After cumulative summing of each load step, absolute cumulative strain maps for each load step were generated. The absolute cumulative strain curves were normalized to extend from the first DVC step until global yield point by interpolation. Since the DVC was performed between two image stacks, the local strains obtained at each loading step represent the total accumulated local strains at the latter acquisition. Average absolute volumetric strain magnitudes, TMDs, and trabecular thicknesses from regions surrounding cracks and from non-crack regions were calculated, analyzed and plotted using custom made MatLab scripts.

**Figure 5:**
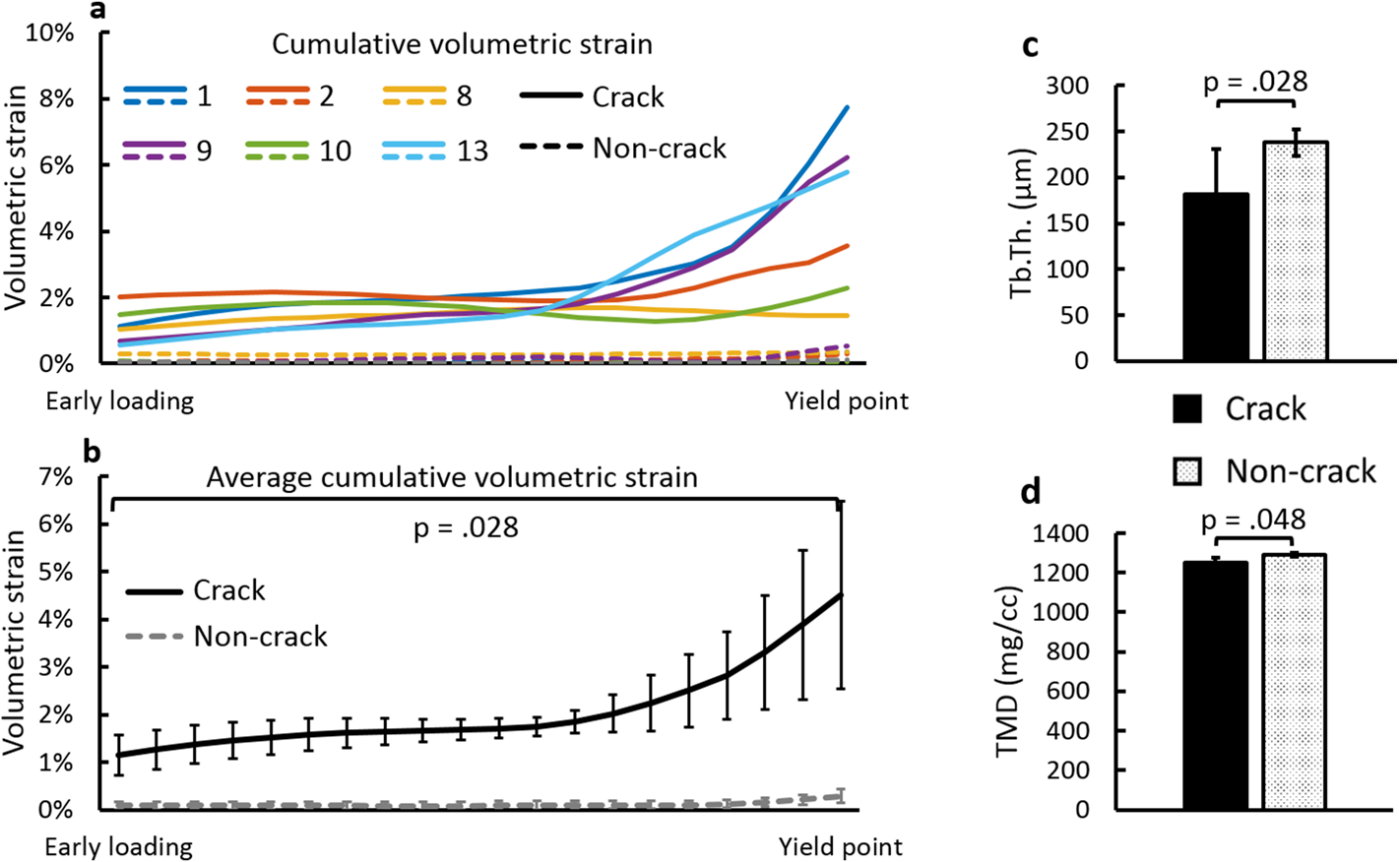
Evolution of cumulative average strains in the crack and non-crack regions. (**a**) Interpolated cumulative absolute volumetric strains for each of the 6 samples, averaged at crack regions (solid lines) and non-crack regions (dashed lines). (**b**) Interpolated average cumulative absolute volumetric strains ± 95% CI of all samples averaged. Error bars are spread over the loading region and do not represent the loading steps. (**c**) Trabecular thickness (Tb.Th.) and (**d**) tissue mineral density (TMD) of crack and non-crack regions when all samples were averaged. *p* values indicate the significance of difference based on Wilcoxon signed ranks test for paired samples (crack regions vs. non-crack regions within each sample). Mean ± 95% CI are shown.

For comparison of the strains between the high-resolution and downscaled data, the binary images of the dilated crack regions were also downscaled using the same factors as the downscaled SR-µCT data. Thus, the strains in the same regions for each voxel size (resolution) image were used for the calculation of the average strains in the crack and non-crack regions during loading. To compare the strain magnitudes in crack regions between the high-resolution and downscaled data, histograms of the strain magnitudes were created for each voxel size image, normalized to the total number of points in the crack regions.

### Statistical analysis

Mean values, standard deviations (SD), and coefficient of variance (CV%) were calculated for all the values derived from the microstructural, mechanical, and strain analyses. Linear regression analyses were performed between microstructural parameters and mechanical properties for all 13 samples by defining mechanical parameter as dependent variables and microstructural parameters as independent variables. Multiple linear regression between combinations of independent variables BV/TV, Tb.Th., and DA and dependent mechanical parameters were also investigated. Additionally, two-tailed Pearson correlation analysis was done on the six samples analyzed by DVC, between averaged local strains at the crack regions measured at the global yield point and BV/TV, TMD, Tb.N., and global yield strain. The cracked and non-cracked regions within each sample were considered related, thus the non-parametric Wilcoxon signed rank test was used to compare strain magnitudes, trabecular thicknesses, and tissue mineral densities. For all analyses, *p* < 0.05 was considered statistically significant. All statistical analyses were done using IBM SPSS statistics (version 25, IBM Corp., Armonk, NY). Additionally, 95% confidence intervals (CI) are displayed in the results figures. To evaluate the effect sizes, 95% CI of the difference between means were calculated.

## Results

### Sub-trabecular resolution strain analysis

The distribution and evolution of strain magnitudes within the trabecular bone was assessed by DVC between two consecutive image stacks. The summed point-by-point local strains within the trabeculae obtained at each loading step represent the total accumulated local strains at the latter acquisition time (Fig. 4). These strain maps clearly display elevated strains in the crack regions.

**Figure 6:**
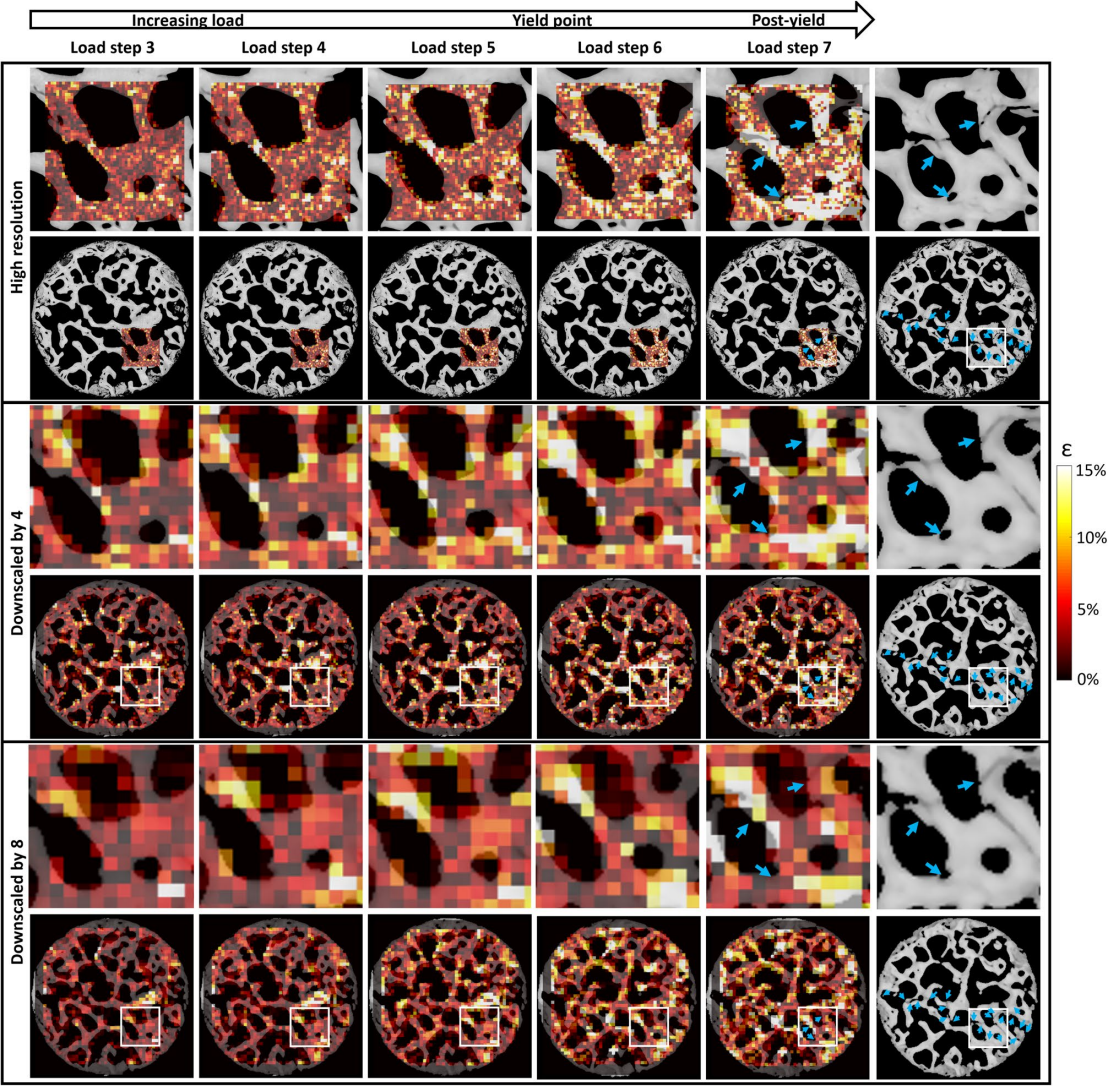
Comparison of strain distributions between high-resolution and downscaled data. DVC absolute cumulative volumetric strain distributions for high-resolution and downscaled data superimposed on the SR-µCT sections on the region of high-resolution DVC and on whole sample section (sample 9). On the second row, the original high-resolution (unfiltered and unmasked) images are shown. Strains from 3rd to 7th loading step are shown where load step 7 is at post-yield and load step 6 shortly after yield-point. Blue arrows indicate cracks and white boxes in the whole sample figures show the ROI of the high-resolution DVC. On the right SR-µCT post-yield images without strains are shown, blue arrows indicate the global fracture line (see Supplementary Fig. 3 for raw volumetric strains between each load step). The direction of loading is perpendicular to the figure plane.

Interpolated averaged strain curves follow the same trend for the 6 analyzed samples, with a constant evolution up until global yield point in the crack regions (Fig. 5a, b). The average ± SD volumetric strains in the crack regions at yield point were 4.5% ± 2.5% and 0.2% ± 0.2% at the non-crack regions (Supplementary Table 1). The crack regions, as well as non-crack regions, contain high (> 10%) strain values locally (Figs. 4 and 6), but these are smoothed out when the strains in the regions are averaged. Throughout the loading, the average volumetric strains inside the crack regions were significantly greater from first DVC analysis step (first image) until yield point (Fig. 5a, b) compared to the non-crack regions. 95% CI of the difference between means varied from [0.6%, 1.5%] at early loading to [2.2%, 6.2%] at the yield point.

### Effect of voxel size on strain magnitudes

To study the effect of the input image voxel size on the DVC-derived strain magnitudes, all high-resolution data were downscaled by factors of 4 and 8. Clear differences in the magnitudes and distributions of the strains before and at failure were observed with different image voxel sizes (Fig. 6 and Supplementary Fig. 3), especially in small cracks regions. In general, the very high local strains were not captured in the small crack regions when the voxel size was increased. Moreover, despite that the overall crack regions were identified with larger voxel size, the possibility to specifically identify the correct trabeculae that cracked were lost (Fig. 6, Supplementary Fig. 3).

**Figure 7:**
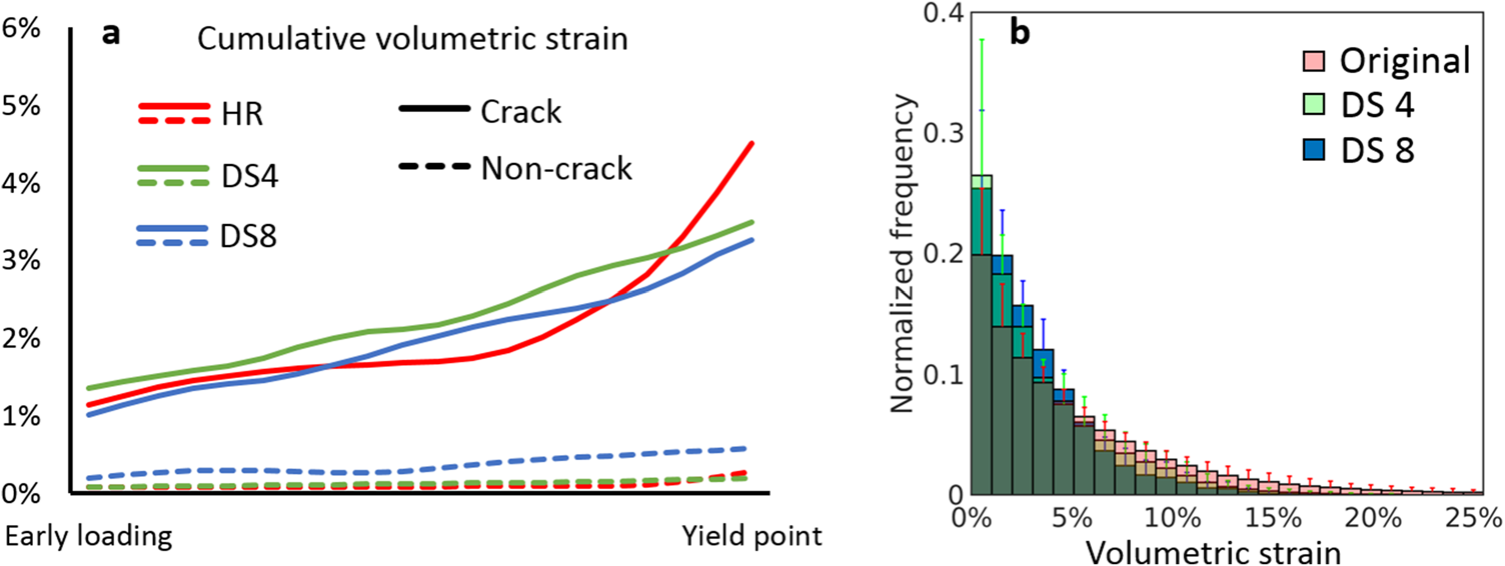
Comparison of strain magnitudes between high-resolution and downscaled data. (**a**) Average interpolated cumulative absolute volumetric strains for high-resolution (HR) and downscaled by a factor 4 (DS4) and 8 (DS8) averaged at the crack (solid line) and non-crack (dashed line) regions. (**b**) Normalized average histograms with SDs of strain magnitude frequencies at crack regions at the load steps before yield point analyzed from the high-resolution and downscaled data.

Histograms of the distributions of the strain magnitude in the crack regions were calculated for each voxel size. At early loading points, the average strain magnitudes in the crack regions between high-resolution and downscaled images are similar, but when approaching the yield point, the high local strains are not captured in the downscaled data analysis (Fig. 7a). The average strains in the downscaled data are higher in the crack regions compared to non-crack regions. However, when comparing the frequency of strain magnitudes in the crack regions, it was not possible to locate the highest strains, evident in the high-resolution data, in the downscaled data (by a factor 4 and 8) (Fig. 7b). Although high (> 10%) strains are evident also in the downscaled data, they are less frequent. Furthermore, because of the lower number of data points in the downscaled data, each bar in Fig. 7b (normalized to the total number of values) represents fewer points with larger voxel size.

### Role of the local trabecular thickness and tissue mineral density in regions to be cracked

The average Tb.Th. and TMD in the crack and non-crack regions were determined (Fig. 5c, d). The average Tb.Th. in the crack regions was 75.9% ± 8.0% [min: 63.3%, max: 85.4%] of the thickness in the non-crack regions (*p* < 0.05, 95% CI of the difference between means [5.5 µm, 107.4 µm]). Also, the average TMD in the crack regions was 97.1% ± 2.3% [min: 94.1%, max: 100.0%] of the TMD in the non-crack regions (*p* < 0.05, 95% CI of difference between means and [15.4 mg/cc, 60.7 mg/cc]). The overall distribution of the TMD is relatively constant throughout the structure and among samples, whereas larger variations in the TMD are noticeable locally through individual trabeculae (Fig. 2d).

### Global microstructural and mechanical properties

The measurement of global microstructure and mechanical properties of the samples confirmed the variability in properties (Supplementary Table 1). The 6 samples chosen for DVC analysis represented well the variation in microstructure and mechanical properties of the thirteen samples tested (Fig. 3 and Supplementary Table 2). Overall, the complete sample set was heterogeneous and showed a large variation in all parameters, with higher variation observed for the mechanical properties compared to the microstructural parameters. The microstructure, especially BV/TV and Tb.Th., correlated significantly with the mechanical properties of the samples (Supplementary Table 3). Moreover, significant positive correlations were found between the local strains at global yield point in crack regions and BV/TV and Tb.N., and high but non-significant correlations with TMD and global yield strain, in the six samples subjected for DVC analysis (Supplementary Fig. 4).

## Discussion

The local strains in human trabecular bone subjected to in situ compressive loading have been evaluated using digital volume correlation analysis based on high-resolution SR-µCT images. This is the first study to utilize DVC on such high-resolution tomographic images (3.6 µm voxel size) of trabecular bone. Compared to earlier studies applying DVC analysis on bone^26–30^, this study presents more extensive analysis of bone strains with sub-trabecular resolution including strain evolution and distribution during loading. The results suggest that the local strains leading to bone tissue yield are generally underestimated in the literature describing whole trabecular structure^10–12, 15^. However, they are in agreement with the high strains measured at isolated single trabeculae^16–21^. Results also indicate that the local strains in crack regions are dependent on the image voxel size at which the strains are measured. Our study is the first to systematically investigate whole trabecular structure at this high resolution (small voxel size), and our findings support that the results from single trabecular tests may be translated to whole trabecular network damage criterion.

The average volumetric strains before fracturing, i.e. at global yield point, were observed to be higher in the crack regions compared to non-crack regions. The average local strains in the crack regions ranged up to 7.7% at the global yield point, which is higher than earlier reported for the trabecular structure^10, 15^. The high localized individual strain values show that the yield strains for bone as a material can be much higher (> 10%) than the average strains in the crack regions (Figs. 4 and 7), which also confirms the data earlier presented for single trabeculae under loading^16–21^. The volumetric strain remained at the range of 0.1–0.5% in the non-crack region. Clear intra-subject and within-subject variability in the strains was observed, which is in agreement with earlier findings for human trabeculae^19^. The difference in the strains between crack and non-crack regions was observed throughout the loading regime. The volumetric strains in the non-cracked trabecular network remained low (< 0.5%) also post-yield, whereas strains in the crack regions started to increase exponentially already before the yield point (Fig. 2). Odgaard et al. reported a similar finding, as they noticed that after a region in the trabecular bone sample failed, the strains stopped increasing in the non-cracked regions^15^. These findings suggest that already from the beginning of the loading, strains start to accumulate more in the regions where the trabeculae will finally break. In this study, the local strains were analyzed with DVC only before the global yield point, and it was manually checked that no cracks had yet occurred, since strain analysis on existing cracks would violate the continuity assumptions resulting in strains that are not real and that would be interpreted as very high strain values.

The results in this study further substantiate that local strains are dependent on the image voxel size at which they are determined, especially in the presence of cracks. The analysis of the high-resolution data in this study provides local strain distributions and magnitudes in higher detail than before, whereas the strains from the downscaled data are comparable to earlier studies. Bay et al. discussed the high-strain issue, as they observed local continuum-level strains reaching 6–7% levels still in the linear elastic region of loading^26^. Based on their findings and the results in this study, the strains may reach locally very high levels (> 10%), which are only detectable with small enough voxel size or when the volume about to crack is large enough for the strains to be captured also with larger voxel size. This suggests that the magnitudes of detected strains and located cracks are dependent on the voxel size, as seen in the average cumulative strain evolutions between different voxel sizes and strain magnitude histograms (Fig. 7). In general, the strains observed in the analysis of the high-resolution (small voxel size) images were towards the magnitude of the strains measured on single trabeculae^16–21^, and the strains observed in the analysis of the downscaled images were towards the magnitude of the strains determined from larger voxel size data when the trabecular network structure was considered^10–12, 15^. Bouxsein et al. recommended a minimum of 3–4 voxels across the thickness of individual trabeculae to minimize the numerical errors when calculating trabecular parameters^48^. Transposed to this study, this requirement was reached for the high-resolution data, with more than 3–4 DVC points obtained across the trabeculae. The criterion was not fulfilled in the downscaled data (1–2 voxels across the cracked trabeculae), indicating that it is not possible to detect the cracks that occur in the sub-trabecular level. Although the overall crack-regions were identified in the downscaled data, the high strain regions do not match perfectly with the high-resolution strain locations or even fail to capture the crack completely, especially in the data downscaled by factor of 8 (e.g. crack in the top-right corner of the high-resolution DVC region in Fig. 6). It has to be noted that it is currently not possible to determine if the high local strains measured in this study from the high-resolution images have reached the converged state. It may be possible that even smaller voxel size could yield higher calculated strains. However, in this study, we have pushed the technological development as far as currently possible, while keeping the sample size on the tissue scale and the imaging times low. In the future, an experimental convergence study of the local strains close to damage would be desired. Moreover, the voxel size was not sufficient for detecting possible yielding inside the material, i.e. within collagen fibers and mineral crystals, which could be present in bone shortly before fracturing^24^. Nevertheless, for development and validation of computational bone damage and fracture models, this aspect is important, since it will affect how to translate experimental findings to computational studies as mesh-size used in the finite element model could be related to the fracture strain threshold.

The locations of cracks seem to be more influenced by the local trabecular thickness than the TMD, although both are reduced at local crack sites (Fig. 5c, d). The trabeculae were thinner in the crack regions, being on average 75.9% of the trabecular thickness of the non-crack regions. Bay et al. similarly noticed that strains concentrated in a less dense part of the trabecular structure^26^. The difference in the TMD was much smaller (97.1%), but was still lower or equal in all crack regions. Also correlation analysis showed that the averaged local strains at crack regions at the global yield point seem to be increased with elevated overall TMD (Supplementary Fig. 4). However, from the averaged structural parameters, Tb.N. and BV/TV seemed to have the highest correlation with the local strains at global yield point (Supplementary Fig. 4). Taken together, these results suggest that the magnitude of both global and local strains needed for the bone to crack are likely to be dependent on the total amount and mineral density of bone, whereas the crack location is controlled by the local trabecular thickness.

In a spongy-like connected structure such as trabecular bone, the structure breaks at its weakest link, which is generally the location where the trabeculae are thinnest. It is likely that locally thicker trabeculae can withstand higher loads than thin trabeculae due to differences in strain energy density. On the opposite, a higher number of trabeculae seems to increase the local strains required to reach yield, which could be interpreted as that it leads to a more even distribution of the strains before cracks appear. To better evaluate the effect of the local trabecular variation of TMD on crack formation, data at the initiation point of the crack would be required. Since the imaging in this study was performed stepwise after each load step (and not continuously), information about the crack initiation point and direction is lacking and would require high-speed imaging. It should be noted that also bone constituents other than mineral density, e.g. collagen fibers and cross-links, play an important role in the bone mechanics, but the characterization of these was not possible with the available data and was not the focus of the study. Furthermore, the structural organization surrounding the crack regions might have an important role in dictating the locations where stresses and strains accumulate. However, as this study focused on the exact regions where cracks were formed, further studies are needed for more detailed analysis of the response of the surrounding regions.

At the global level, i.e. for a whole compressed sample, the BV/TV and average Tb.Th. are the most important characteristics that provide bone with its mechanical properties (Supplementary Tables 3 and 4). This has also been observed earlier^51, 52^ and highlights that the amount of material is the most important contributor to strength. Overall, the values of the microstructural and mechanical parameters in this study are in line with earlier studies testing femoral head trabecular bone^51, 53^.

As an interesting secondary finding, the data suggests that trabecular bone seems to be able to withstand momentarily local high strains (~ 10%) without fracturing (for example, see Supplementary Fig. 2), i.e. locally the structure exhibits alternating positive and negative volumetric strains over the loading, observed in all the samples in this study. Hypothetically, this alternation in the strains is most probably due to redistribution of strains elsewhere in the trabecular network. However, when the region where the high strains accumulate is large enough, relative to the thickness of the trabecula, a crack occurs due to higher strain energy density (Supplementary Fig. 2). This could be important to account for when establishing fracture criteria in computational bone damage models. This finding also supports the result that trabecular thickness is crucial in defining a crack location; thinner trabeculae are more likely to be subjected to high strains over their entire thickness and eventually fail. Still, trabecular thickness is most likely not the only factor affecting the location of the crack since local material properties, e.g. mineral density and composition, play a crucial role in fracture resistance. As trabecular bone tissue is largely heterogeneous in terms of structure and composition, the fracture resistance varies throughout the tissue. However, based on the current data it is not possible to evaluate the combined or separate effects of structure and composition on the crack initiation. Moreover, in this study TMD was the only investigated compositional property while other compositional properties, e.g. collagen content, also significantly contribute to the mechanical properties of bone^54^. It has to be noted that although high local strains might not directly result in cracking of the trabecula, they might induce micro-cracks that are not visible with the image voxel size used in this study. These results need further careful verification to understand its role contra the global fracture properties of trabecular bone.

Increasing interest towards SR-µCT in tissue imaging has triggered the exploration of an important side effect of the technique: radiation damage. In particular, it is crucial to consider the radiation dose inflicted by image acquisition, when analyzing the mechanical properties of biological tissues. It has recently been shown that no major changes in the mechanical integrity of bone are evident with radiation doses below 33 kGy, whereas the post-yield plastic behavior is completely absent with doses above 70 kGy^42, 44^. These values can be taken as guidelines, but, generally, the radiation doses must be minimized for optimal results, i.e. to avoid damaging the collagen in the tissue that contribute to its mechanical integrity. In this study, the estimated maximum doses received by the samples throughout the tests did not exceed 28 kGy (including sample alignment), thus, no major detrimental effect to the elastic and early post-yield behavior due to the imaging was expected. To minimize the dose in future studies, this value can be significantly reduced by using a more optimized sample alignment and image acquisition procedure^41^.

The accuracy and precision of the DVC technique was evaluated using repeated measurements on two overlapping scans in between the acquisition of which the sample had been vertically moved; it would have been preferable to use two repeated images of the same region. This translation may cause some movement artifact at the micrometer level, e.g. tilting, and add detector related artifacts as the repeated region of interests were not obtained from the same detector position. Moreover, beam intensity fluctuations from bottom to the top of the image stack, although taken into account in the reconstructions, might have an effect on the subtle changes in contrast inside the trabeculae. However, as these limitations added noise to the images, they lead to a measurement of a rather maximal error of the DVC procedure. Despite this, the measured DVC accuracy and precision values were acceptable and comparable to previous studies with similar DVC parameters^32–35^ and were one to two orders of magnitude lower than strains measured in the crack regions during loading. However, they were of the same order of magnitude as the average strains in the non-cracked regions, which could limit the validity of these strains. It has to be noted that these limitations in determination of accuracy and precision do not fully apply to the loading scans, in which the displacement of the sample was limited, thus the analyzed field of view stayed mostly constant in the detector position.

All our samples were extracted parallel to the femoral neck. Thus, the sample orientation and primary alignment was highly consistent. Still, a small natural variation in the DA and primary orientation of the trabecular network within the femoral heads occurred, and in some samples the loading axis was slightly tilted compared to the main axis of orientation. This may to some extent affect the measured mechanical properties, but are assumed to be within a small range.

The Saint–Venant’s principle suggests that to have a region with uniform stress, the length has to be greater than the diameter of the sample. This is a limitation in this study, but as stated above already, to image a whole sample of this length, at the defined high-resolution, required three stacked scans due to the height of the synchrotron beam. Thus, longer samples would have required an undesirable compromise on image resolution. Due to the limited region of the sample that was imaged during loading and because of the refined region chosen for the DVC analysis (due to required heavy computations) not all trabeculae about to crack were captured. However, based on the image data, it seems that the whole structure collapses in a catastrophic manner (Fig. 6 and Supplementary Fig. 3); throughout the samples, majority of the trabeculae fractured approximately at the same loading phase, immediately after global yield point. This can be assumed to hold true throughout the sample, with a possible exception at boundaries close to the compression plates, and should have negligible effects on the outcome of this study.

## Conclusions

We have presented the first study of sub-trabecular strain magnitudes, evolution, and distribution in trabecular human bone considering also the structure of the trabecular network. This study shows that the local tissue strains in regions where cracks are about to occur are in agreement with the high strains observed in isolated single trabeculae and demonstrates that the observed strain magnitudes are dependent on the input image voxel size, which provides a potential explanation of why earlier studies with larger image voxel sizes and considering the structure of the trabecular network have presented lower strains. At a local level, the trabecular microstructure, especially trabecular thickness, appears to be the most prominent factor in determining where the trabecular structure will break. In addition, these findings will serve as further validation for computational trabecular bone damage models.

## Supplementary information

Supplementary information
