# Supplementary material for: Sub-trabecular strain evolution in human trabecular bone
Source: Sci Rep. 2020 Aug 14;10:13788. doi: 10.1038/s41598-020-69850-x (PMC7429852; doi:10.1038/s41598-020-69850-x)
Supplement: Supplementary file 1 — Supplementary information [file 41598_2020_69850_MOESM1_ESM.pdf]

Sub-trabecular strain evolution in human trabecular bone

Mikael J Turunen, Sophie Le Cann, Erika Tudisco, Goran Lovric, Alessandra Patera, Stephen A Hall, Hanna Isaksson

The full article is available at https://doi.org/10.1038/s41598-020-69850-x.

SUPPLEMENTARY MATERIAL

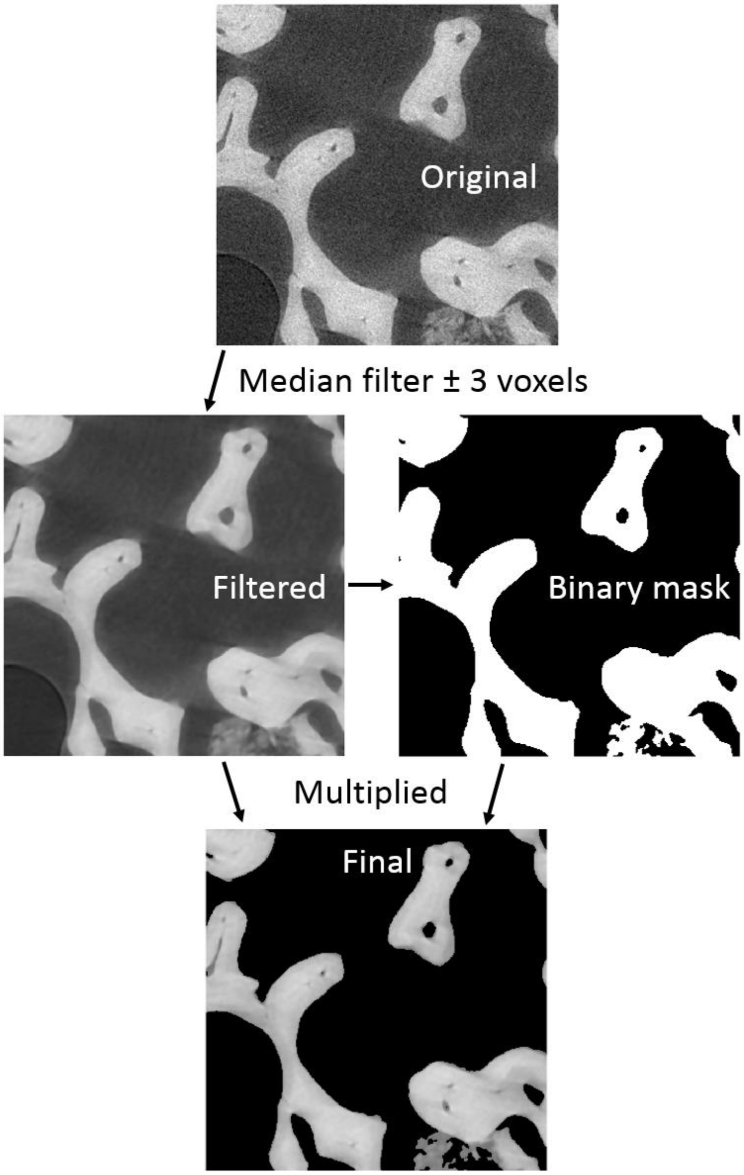

Supplementary Figure 1. Image pre-processing. µCT images were pre-processed by using a median filter of ± 3 voxels and subsequently a binary mask was defined. The final image for DVC analysis was obtained by multiplying the filtered image with the binary mask.

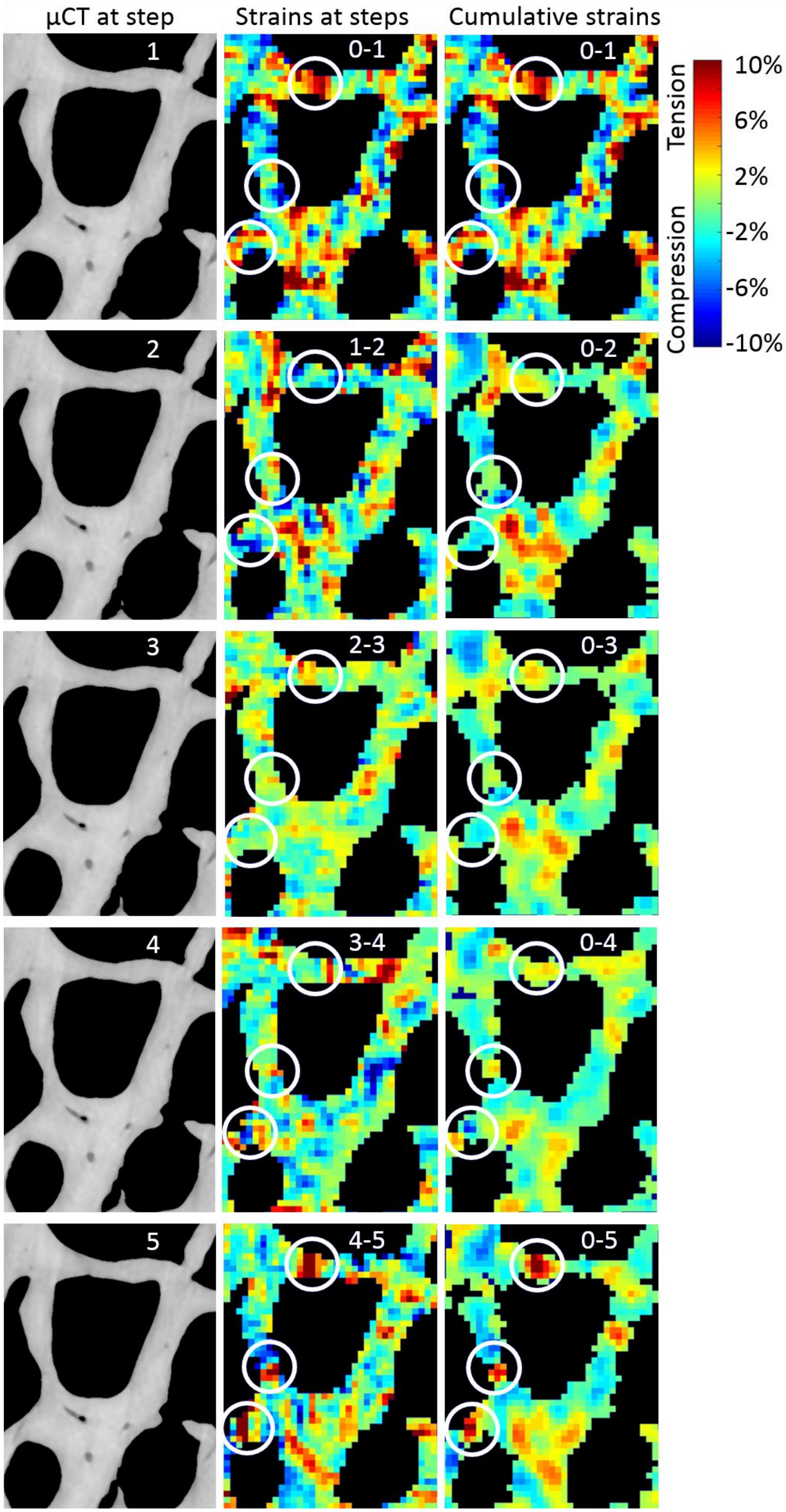

Supplementary Figure 2. Compressive and tensile strain distribution evolution during loading. Example of SR-µCT 2D slices from 1st to 5th imaging step (left column), the respective strain distributions in each DVC analysis step (middle column,) and cumulative strains (right column) from the sub-region of sample 1. The crack locations in each strain sub-image are circled in white. High localized ~10% strains are evident in the individual DVC analysis steps but are canceled out during the loading in the non-crack regions. Note the high tensile strains already at the first DVC analysis step at the topmost crack region, which are redistributed during the loading, but finally lead to cracks at those locations. The global yield point for this sample is just before load step 5, where some cracks are barely visible.

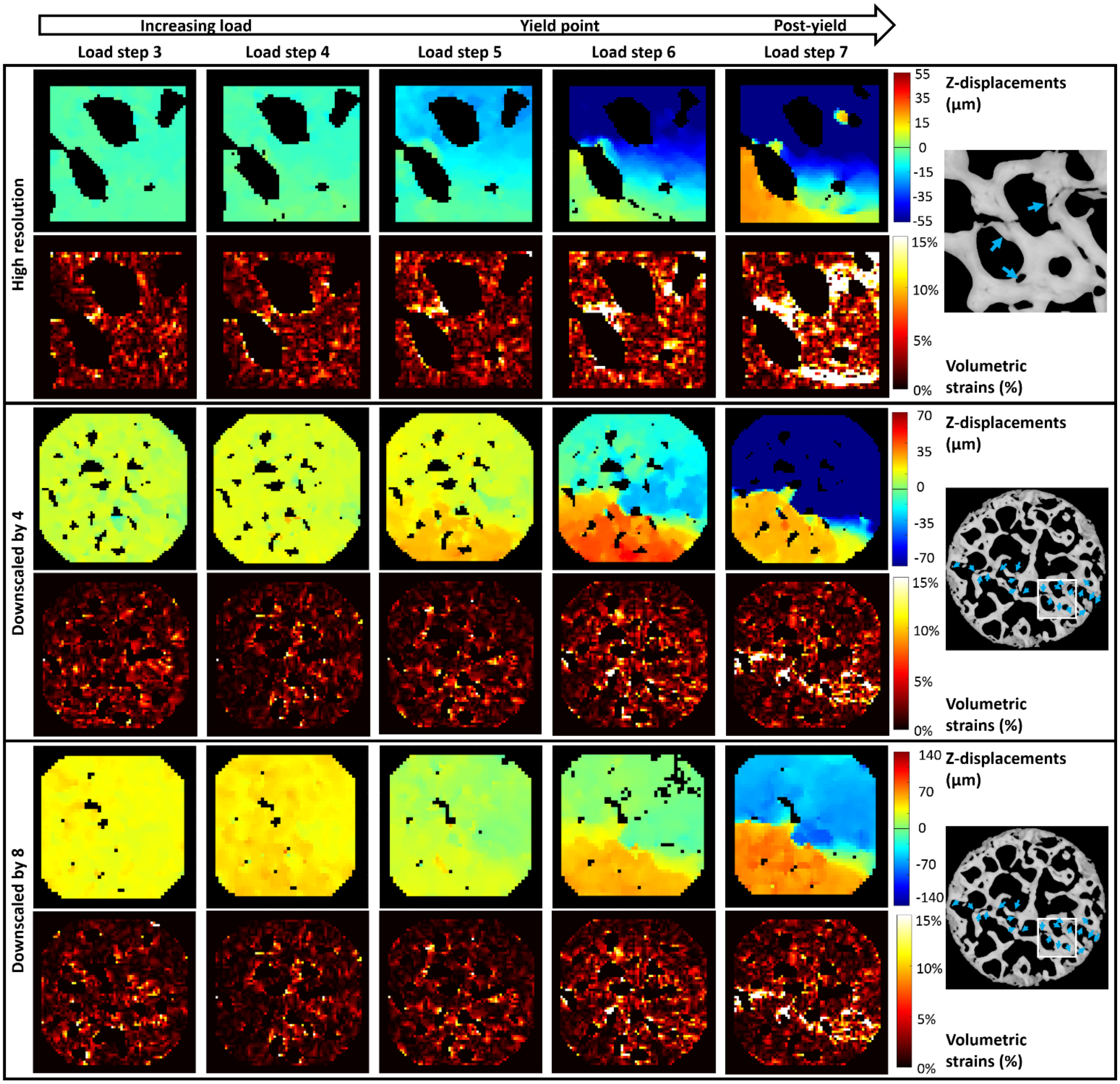

Supplementary Figure 3. Z-displacements and step-wise volumetric strains. Raw (filtered) z-displacement and volumetric strain maps between two consecutive load-steps from DVC analysis for high-resolution and downscaled data for same sample section as presented in Figure 6. In this figure, all data points are shown (without removing correlation coefficient < 0.95). Displacements volumetric strains from 3rd to 7th loading step are shown where step 7 is at post-yield and step 6 shortly after yield-point. The catastrophic collapse of the structure is clearly seen in the 6th and 7th load step. Displacements are presented in voxels and volumetric strains in percentage. Z-displacement is in the direct of loading, perpendicular to the figure plane. On the right SR-µCT post-yield images without strains are shown, blue arrows indicate the global fracture line.

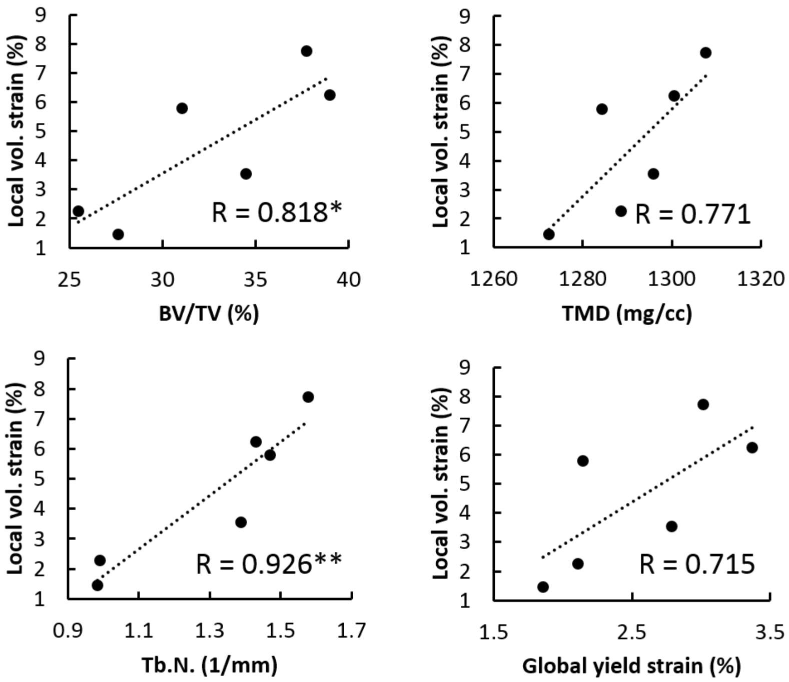

Supplementary Figure 4. Correlations between local strains from DVC in crack regions obtained at the global yield point and global parameters. Scatterplots showing linear correlations between averaged local volumetric strains at crack regions at yield point and DVC region structural parameters (BV/TV, TMD, and Tb.N.) and global yield strains in the DVC analyzed samples. Linear regression coefficients (R) are indicated with significances * $p < 0.05$, ** $p < 0.01$.

Supplementary Table 1. Apparent modulus and global yield strain, as well as microstructural parameters and local trabecular strains at yield point for the sub-regions used in DVC analysis. Bone volume fraction (BV/TV), averaged tissue mineral density (TMD), as well as averaged trabecular thickness (Tb.Th.), trabecular number (Tb.N.), and degree of anisotropy (DA) of the sub-regions are presented. Averaged cumulative volumetric strains at yield point for the crack and non-crack regions are listed. Means, SDs, and CV% are shown for each variable.

	Whole sample		DVC analysis region						
			Microstructure					Local strains at global yield point	
**Sample**	**Apparent modulus MPa**	**Yield strain %**	**BV/TV %**	**TMD mg/cc**	**Tb.Th. μm**	**Tb.N. 1/mm**	**DA AU**	**crack %**	**non-crack %**
1	300	3.0	37.7	1308	239	1.58	2.11	7.7	0.1
2	441	2.8	34.5	1296	248	1.39	2.82	3.5	0.3
8	326	1.9	27.6	1272	281	0.98	2.53	1.5	0.3
9	487	3.4	39.0	1301	273	1.43	1.38	6.2	0.5
10	464	2.1	25.5	1289	257	0.99	1.90	2.3	0.1
13	401	2.1	31.0	1284	211	1.47	2.16	5.8	0.1
**mean**	**403**	**2.5**	**32.6**	**1292**	**251**	**1.31**	**2.15**	**4.5**	**0.2**
**SD**	**76**	**0.6**	**5.4**	**13**	**25**	**0.26**	**0.50**	**2.5**	**0.2**
**CV%**	**18.8**	**23.4**	**16.7**	**1.0**	**10.0**	**19.6**	**23.2**	**54.6**	**79.3**

Supplementary Table 2. The microstructure and mechanical properties of all samples. Gender (M = male, F = female) and age of the cadavers, and microstructural and mechanical properties of all sample plugs. For microstructure, bone volume fraction (BV/TV), averaged tissue mineral density (TMD), as well as averaged trabecular thickness (Tb.Th.), trabecular number (Tb.N.), and degree of anisotropy (DA). For mechanical properties, apparent modulus, ultimate and yield stresses, ultimate and yield strains (delta strains), and toughness are presented. Additionally, means, SDs, and CV% among all samples are shown for each variable. * denotes the samples analyzed with DVC.

			**Microstructure**				**Mechanical properties**						
**Sample**	**Gender**	**Age**	**BV/TV**	**Tb.Th.**	**Tb.N.**	**DA**	**Ext. Stiffness**	**Apparent modulus**	**Ultimate stress**	**Yield stress**	**Ultimate strain**	**Yield strain**	**Toughness**
			**%**	**µm**	**1/mm**	**AU**	**N/mm**	**MPa**	**MPa**	**MPa**	**%**	**%**	**MPa**
1*	M	82	35.9	240	1.50	2.42	1855	300	9.8	8.5	3.8	3.0	0.21
2*	M	54	36.4	258	1.41	2.65	2781	441	11.9	11.4	3.1	2.8	0.22
3	M	49	39.9	272	1.47	2.06	2693	430	11.1	10.5	3.1	2.6	0.20
4	M	46	39.2	260	1.51	2.18	2689	410	11.7	11.1	3.3	2.9	0.22
5	M	59	25.4	187	1.35	2.20	1878	298	5.0	4.8	2.1	1.8	0.07
6	M	74	47.2	250	1.89	1.39	2912	451	12.0	11.6	3.0	2.8	0.20
7	M	78	42.4	251	1.69	1.91	2568	402	9.7	9.3	3.1	2.5	0.18
8*	F	58	28.0	243	1.15	2.65	2055	326	6.1	5.4	2.5	1.9	0.10
9*	M	39	45.2	297	1.52	1.24	3113	487	16.4	15.5	4.0	3.4	0.37
10*	M	21	31.8	287	1.11	1.66	2802	464	9.8	9.8	2.3	2.1	0.12
11	M	52	39.8	239	1.67	2.73	2947	477	11.7	10.8	3.0	2.5	0.21
12	M	64	36.4	231	1.58	1.54	1531	235	7.2	6.3	3.9	2.9	0.17
13*	M	60	32.0	227	1.41	2.13	2515	401	8.0	7.8	2.3	2.1	0.11
**Mean**		**56.6**	**36.9**	**249**	**1.48**	**2.06**	**2488**	**394**	**10.0**	**9.4**	**3.0**	**2.6**	**0.18**
**SD**		**16.5**	**6.4**	**28**	**0.21**	**0.49**	**494**	**79**	**3.0**	**2.9**	**0.6**	**0.5**	**0.08**
**CV%**		**29.1**	**17.4**	**11.2**	**14.2**	**23.8**	**19.9**	**20.1**	**29.9**	**30.9**	**20.1**	**18.4**	**42.1**

Supplementary Table 3. Linear regression coefficients (R) between trabecular bone microstructure: bone volume fraction (BV/TV), trabecular thickness (Tb.Th.), trabecular number (Tb.N.), and degree of anisotropy (DA), and mechanical properties: extrinsic stiffness, apparent modulus, ultimate and yield stresses, ultimate and yield strains, and toughness for all 13 samples. ** $p < 0.01$ and * $p < 0.05$.

Linear regression		Ext. Stiffness N/mm	Apparent modulus MPa	Ultimate stress MPa	Yield stress MPa	Ultimate strain %	Yield strain %	Toughness MPa
**BV/TV**	**%**	0.593*	0.535	0.816**	0.802**	0.649*	0.762**	0.801**
**Tb.Th.**	**µm**	0.669*	0.670*	0.773*	0.788*	0.335	0.449	0.658*
**Tb.N.**	**1/mm**	0.225	0.159	0.410	0.383	0.530	0.584*	0.466
**DA**	**AU**	-0.171	-0.133	-0.332	-0.356	-0.323	-0.424	-0.351

Supplementary Table 4. Multiple linear regression model coefficients (R) between combinations of bone volume fraction (BV/TV), trabecular thickness (Tb.Th.), and degree of anisotropy (DA), and mechanical properties: extrinsic stiffness, apparent modulus, ultimate and yield stresses, ultimate and yield strains, and toughness for all 13 samples. ** $p < 0.01$ and * $p < 0.05$.

Multiple linear regression model	Ext. Stiffness	Apparent modulus	Ultimate stress	Yield stress	Ultimate strain	Yield strain	Toughness
**BV/TV + Tb.Th + DA**	0.746	0.730	0.912**	0.907**	0.650	0.766*	0.845**
**BV/TV + Tb.Th**	0.721*	0.698*	0.903**	0.902**	0.649	0.763*	0.842**
**BV/TV + DA**	0.606	0.552	0.819**	0.802**	0.649	0.766*	0.802**
